# Genetic variants in genes related to inflammation, apoptosis and autophagy in breast cancer risk

**DOI:** 10.1371/journal.pone.0209010

**Published:** 2019-01-02

**Authors:** Johanna M. Schuetz, Anne Grundy, Derrick G. Lee, Agnes S. Lai, Lindsay C. Kobayashi, Harriet Richardson, Jirong Long, Wei Zheng, Kristan J. Aronson, John J. Spinelli, Angela R. Brooks-Wilson

**Affiliations:** 1 Canada’s Michael Smith Genome Sciences Centre, BC Cancer Agency, Vancouver, British Columbia, Canada; 2 CRCHUM (Centre de recherche du Centre hospitalier de l’Université de Montréal), Montreal, QC, Canada; 3 Department of Social and Preventive Medicine, Université de Montréal, Montreal, QC, Canada; 4 Department of Cancer Control Research, British Columbia Cancer Agency, Vancouver, BC, Canada; 5 Department of Mathematics, Statistics, and Computer Science, St. Francis Xavier University, Antigonish, NS, Canada; 6 Harvard Center for Population and Development Studies, Harvard T. H. Chan School of Public Health, Cambridge, MA, United States of America; 7 Department of Oncology, Lombardi Comprehensive Cancer Center, Georgetown University, Washington, DC, United States of America; 8 Department of Public Health Sciences, Queen's University, Kingston, ON, Canada; 9 Division of Cancer Care and Epidemiology, Cancer Research Institute, Queen’s University, Kingston, ON, Canada; 10 Vanderbilt Epidemiology Centre, Vanderbilt University Medical Center, Nashville, TN, United States of America; 11 School of Population and Public Health, University of British Columbia, Vancouver, BC, Canada; 12 Department of Biomedical Physiology and Kinesiology, Simon Fraser University, Burnaby, BC, Canada; University of South Alabama Mitchell Cancer Institute, UNITED STATES

## Abstract

**Background:**

Inflammation contributes to breast cancer development through its effects on cell damage. This damage is usually dealt with by key genes involved in apoptosis and autophagy pathways.

**Methods:**

We tested 206 single nucleotide polymorphisms (SNPs) in 54 genes related to inflammation, apoptosis and autophagy in a population-based breast cancer study of women of European (658 cases and 795 controls) and East Asian (262 cases and 127 controls) descent. Logistic regression was used to estimate odds ratios for breast cancer risk, and case-only analysis to compare breast cancer subtypes (defined by ER/PR/HER2 status), with adjustment for confounders. We assessed statistical interactions between the SNPs and lifestyle factors (smoking status, physical activity and body mass index).

**Results and conclusion:**

Although no SNP was associated with breast cancer risk among women of European descent, we found evidence for an association among East Asians for rs1800925 (*IL-13*) and breast cancer risk (OR = 2.08; 95% CI: 1.32–3.28; *p* = 0.000779), which remained statistically significant after multiple testing correction (*p*_*adj*_ *=* 0.0350). This association was replicated in a meta-analysis of 4305 cases and 4194 controls in the Shanghai Breast Cancer Genetics Study (OR 1.12, 95% CI: 1.03–1.21, *p* = 0.011). Further, we found evidence of an interaction between rs7874234 (*TSC1*) and physical activity among women of East Asian descent.

## Introduction

Chronic inflammation has been linked to cancer development [1], and can influence breast cancer through many mechanisms [2]. One way that chronic inflammation can contribute to cancer is through persistent damage to cells and their components such as DNA. Apoptosis and autophagy are two of the main processes that respond to the consequences of damage caused by inflammation. Proteins involved in inflammation and apoptosis are associated with the invasiveness of breast cancer lines [3] and the maintenance of stem cell properties [4].

Lifestyle factors such as smoking introduce sources of inflammation to breast tissue. Several studies suggest a causal association between lifestyle factors and breast cancer susceptibility or progression. For example, in premenopausal women, obesity can affect hormone-independent breast cancer through chronic inflammation of the breast adipose tissue [5]. In addition, inherited factors could aggravate chronic damage from inflammation, or alternately offset damage and thus protect tissues from inflammation. Genes involved in inflammation and apoptosis have previously been associated with cancer prognosis, [6,7] as well as susceptibility [8–10].

Breast cancers may be classified by pathologists using hormone receptor status (the estrogen receptor [ER] and progesterone receptor [PR]), or HER2 growth receptor status. Some associations of different genes to risk are limited to only certain tumour subtypes [7,11], which hint at etiological and biological differences between subtypes and suggest a benefit in examining the subtypes separately when enough samples and information are available.

The objective of this study was to replicate previously reported associations of polymorphisms in genes related to inflammation, apoptosis and autophagy with risk of breast cancer in women of European and East Asian descent. Within these three gene categories, we included additional genes previously shown to have an effect on cancer. We also tested for statistical interactions of these genes with lifestyle factors that could affect inflammation, including body mass index (BMI), physical activity and smoking status. Finally, we carried out case-only analyses to examine whether these SNPs may have breast tumour subtype specific effects.

## Materials and methods

### Samples and SNPs

This study was approved by the joint Clinical Research Ethics Board of the University of British Columbia and BC Cancer. All participants gave written informed consent.

In brief, the Canadian Breast Cancer Study, a population-based case-control study, invited women with *in situ* or invasive breast cancer, diagnosed from April 2005 to May 2009 [12]. Women were aged 20 to 80 and lived in Metro Vancouver, British Columbia (BC) or Kingston, Ontario regions of Canada. In Vancouver, women were recruited through the BC Cancer Registry, and age-frequency matched controls were recruited through the Screening Mammography Program of BC. In Kingston, cases and controls were recruited from the Hotel Dieu Breast Assessment Program. DNA was obtained through a blood (91%) or saliva sample (9%). Additional information (including menopausal status, ethnicity, and lifestyle characteristics) was obtained through a questionnaire, either by computer assisted telephone interview (22.2%) or self-administered (77.8%).

Fifty-two genes related to apoptosis, autophagy and inflammation were studied (**Table 1**), eight of which had previously identified tagSNPs [13]. Thirty-eight genes were tagged using Tagger [14] in Haploview [15] with HapMap CEU population data (**Table 1**). Additional SNPs were selected for replication of findings from other studies; this resulted in addition of SNPs for 10 candidate genes, and 7 genes with only replication SNPs (**Table 1**). Genotyping was done as part of an Illumina Golden Gate Assay (San Diego, CA, USA) at the Genome Quebec / McGill University Innovation Centre.

**Table 1:** Genes studied.

Category	Gene	Tagged	Replication SNPs	Reference
Apoptosis	*BAX*	X		
	*BBC3/PUMA*	X		
	*BMI1*	X		
	*FAS*	X		
	*MDM2*	X		
	*MIR125A*	X		
	*MIR145*	X		
	*miR15a*	X		
	*MIR206*	X		
	*MIR26A1*	X		
	*MIR30A*	X		
	*MIRN155*	X		
	*MIRN155*	X		
	*PMAIP1/NOXA*	X		
	*RFWD2*	X		
	*SKP2*	X		
Apoptosis & Autophagy	*BCL2*	X		
	*BECN1*	X		
	*MDM2*		rs2279744 & rs937282	Gansmo et al. 2015, PubMed ID 26471763
	*pre-miRNA-27a*		rs895819	Yang et al. 2010, PubMed ID 19921425
	*TP53*	X		
Autophagy	*AKT1*	X		
	*ATG16L1*		rs2241880	In Crohn's disease: Grant et al. 2008, PubMed ID 18366306
	*DRAM*	X		
	*FKBP1A*	X		
	*FLJ20294*	X		
	*FRAP1*	X		
	*KIAA0226*	X		
	*KIAA0831*	X		
	*LKB1/STK11*	X		
	*PARK2*	X		
	*PTEN*	X		
	*TSC1*	X	rs7874234	Mehta et al. 2011, PubMed ID 20658316
	*TSC2*	X		
H2AFX-related	*H2AFX*	X		
	*YY1*	X		
Inflammation	*CASP8 Caspase-8*	*	rs1045485	Reviewed role in inflammation: Loza et al. 2007, PubMed ID 17940599
	*CFH Complement factor H*	*	rs1061170	Reviewed role in inflammation: Loza et al. 2007, PubMed ID 17940599
	*CTLA4*	*	rs3087243	Reviewed role in inflammation: Loza et al. 2007, PubMed ID 17940599
	*IFIH1 Mda-5*, *Helicard*	*	rs1990760	Reviewed role in inflammation: Loza et al. 2007, PubMed ID 17940599
	*IL-13*		rs1800925	Erdei et al. 2010, PubMed ID 20418110
	*IL-4*		rs2243248	Erdei et al. 2010, PubMed ID 20418110
	*IL-10*	X		
	*IL-6*	X		
	*IL1B*	X		
	*IL23R*	X		
	*IL4R*	*	rs1801275	Reviewed role in inflammation: Loza et al. 2007, PubMed ID 17940599
	*INF gamma*		rs2069705	Erdei et al. 2010, PubMed ID 20418110
	*IRF5*	*	rs2004640	Reviewed role in inflammation: Loza et al. 2007, PubMed ID 17940599
	*MAPK14*	X		
	*PTPN22*	*	rs2476601	Reviewed role in inflammation: Loza et al. 2007, PubMed ID 17940599
	*SEPS1*		rs28665122	Role in inflammation: Curran et al. 2005, PubMed ID 16227999
	*TGFB1*	*	rs1982073	Reviewed role in inflammation: Loza et al. 2007, PubMed ID 17940599
	*TNF-alpha*	*	rs1800629 & rs361525	Reviewed role in inflammation: Loza et al. 2007, PubMed ID 17940599

* Tag from Loza et al 2007

Quality control was performed by the authors as described in [12], using Illumina Genome Studio v2011.1 (San Diego, CA, USA), PLINK v1.07 [16], GRR [17] and Microsoft Excel 2007 (Redmond, WA, USA). Of the 221 SNPs selected, 15 (7%) failed quality control for the following reasons: failure in Illumina’s built-in controls (n = 6), GenTrain Score<0.4 (n = 2), poor clusters (n = 4), monoallelic (n = 2), and call rate <0.95 (n = 1) (**S1 Table**).

For this analysis, only individuals of European and East Asian descent were included (**Table 2**). Hormone receptor status (ER, PR and HER2 status) was determined using immunohistochemistry and fluorescence in situ hybridization, as previously described [18], and tumour marker status was dichotomized as “present” or “absent”, as opposed to the cut-off levels of staining used for clinical purposes.

**Table 2:** Characteristics of participants whose samples passed quality control.

		European		East Asian	
		Controls	Cases	Controls	Cases
**Region**					
	Vancouver, Canada	721	573	127	262
	Kingston, Canada	74	85	0	0
**Age group (years)**					
	20–29	0	2	0	1
	30–39	5	20	0	17
	40–49	202	148	53	97
	50–59	271	212	34	68
	60–69	201	169	30	46
	70+	116	107	10	33
**Menopausal Status**					
	Premenopausal	268	189	66	107
	Postmenopausal	525	446	61	137
**Hormone Receptor Status**					
	ER and/or PR positive	0	477	0	174
	ER/PR negative	0	100	0	29
**HER2 Status**					
	Negative	0	532	0	206
	Positive	0	105	0	38
**BMI**					
	<25	458	308	103	200
	25+	336	329	24	44
**Smoking Status**					
	Non-smoker	406	295	111	220
	Smoker	388	342	16	24
**Physical Activity**					
	<89.6 MET hrs/wk	383	329	77	137
	>89.6 MET hrs/wk	401	293	49	101
**Total**		795	658	127	262

### Statistical analysis

Statistical analyses were carried out in SVS (Golden Helix, Inc., Bozeman, MT, USA) and R (version 3, R Foundation for Statistical Computing, Vienna, Austria) [19]. Logistic regression was carried out in European and East Asian women separately, with the inclusion of terms for age group, region of collection, menopausal status, and two interaction terms: menopausal status—age group, and menopausal status–region of collection. Multiple testing correction was applied using the false discovery rate method (FDR) [20] in two stages, as described previously [21]. Briefly, FDR was applied within each gene first, to give a corrected *p*-value for each test. The lowest *p*-value was taken to represent each gene, and finally another round of FDR was applied to yield corrected *p*-values across all the genes tested. This allows correction for the different number of SNPs in each gene, as well as the number of genes tested. FDR-corrected *p*-values of less than 0.05 were taken to indicate an association of the SNP with breast cancer.

SNPs that had *p*<0.05 before multiple testing correction were tested for interaction with being overweight/obese at the time of diagnosis (defined as BMI≥25), smoking status (non-smokers vs. pack-years>0) and total lifetime physical activity. Total lifetime physical activity measurements were measured as previously described [22,23] and included leisure-time, household, and occupational activities of moderate-to-vigorous intensity, summarized using metabolic equivalent (MET) scores (which are the ratio of the calculated metabolic rate for a specific activity compared to resting metabolic rate). In interaction analyses described here and previous work [24], active women were those >89.6 MET hrs/wk (the mean value among controls, which can be approximated by running at a fast pace for a bit under an hour per day). If there was a significant interaction, odds ratios for that SNP were computed within each stratum. Interaction analyses were done separately for Europeans and East Asians. Additional analysis to compare the effect of rs7874234 with regards to lifetime physical activity (in East Asian women) was conducted using StatPlus:mac (AnalystSoft Inc., Walnut, CA, USA).

Finally, logistic regression using only the cases was utilized to examine heterogeneity by hormone (ER/PR) receptor status and HER2 status using the categories indicated in **Table 2**. Analysis was only done for SNPs with *p*<0.05 before multiple testing correction. We did not have samples in each genotype category for the HER2 status case-only analysis in East Asian samples, as the minor allele frequencies were too low. Multiple-testing correction was applied to all SNPs that underwent case-only analysis. Additional analysis to compare the effect of rs7236090 in different hormone receptor status groups in East Asian women was also done using StatPlus:mac.

### Replication analysis

Participants in the Shanghai Breast Cancer Genetics Study (SBCGS) are from four population-based studies: the Shanghai Breast Cancer Study (SBCS), the Shanghai Women's Health Study (SWHS), the Shanghai Breast Cancer Survival Study (SBCSS) and the Shanghai Endometrial Cancer Study (SECS, controls only). Detailed descriptions of these studies have been published elsewhere [25]. Briefly, the SBCS included two recruitment phases, SBCS-I (1996–1998) and SBCS-II (2002–2005). Breast cancer cases were identified through a rapid case ascertainment system and the Shanghai Cancer Registry. Controls were randomly selected from the general female population using the Shanghai Resident Registry and were frequency matched to cases by age. The SBCSS included newly diagnosed breast cancer cases ascertained via the Shanghai Cancer Registry between 2002–2006. The SECS was conducted from 1997–2003; only community controls from the SECS were included in the SBCGS. The SWHS is a prospective cohort study recruited from 1996–2000. The cohort has been followed by a combination of record linkage and active follow-ups to identify cause-specific mortality and cancer incidence by site. These studies are conducted among Chinese women in Shanghai using very similar protocols for data and sample collection: 2,511 breast cancer cases and 2,135 controls were genotyped using an Affymetrix 6.0 array; another 1,794 cases and 2,059 controls were genotyped using the Illumina Multi-Ethnic Genotyping Array. Data quality control procedures were carried out as previously described [25], followed by imputation with 1000 Genomes Project Phase 3 as reference. Logistic regression was performed under an additive model, with adjustment for age and the top five principal components. Data for SNPs rs1800925 and rs7874234 were extracted.

## Results

Characteristics of the cases and controls are summarized in **Table 2.** Briefly, quality control measures resulted in data for 922 controls and 920 cases for 206 SNPs (**S1 Table**). Following logistic regression analysis in Europeans (658 cases and 795 controls), 3 SNPs had p<0.05 before FDR correction and none had *p*<0.05 after multiple testing correction (**S2 Table**): (i) rs6676805 (*RFWD2*) (OR = 0.730 (95% CI: 0.600–0.889) *p* = 0.00158 and after correction *p*_*adj*_ = 0.213), (ii) rs617078 (*RFWD2*) (OR = 0.799 (95% CI: 0.681–0.938) *p* = 0.00585) and (iii) rs8063461 (*TSC2*) (OR = 0.823 (95% CI 0.711–0.963) *p* = 0.00139 and after correction *p*_*adj*_ = 0.960). There was no evidence for an interaction of any of the three SNPs with excess weight, smoking status, or exercise (**S2 Table**). There was also no evidence for differential effects depending on the tumour subtype as determined by hormone receptor status (**S2 Table**).

Among East Asian women (262 cases and 127 controls), 7 SNPs had *p*<0.05 before multiple testing correction (**Table 3**). Following FDR correction, the SNP rs1800925 (*IL-13*) was associated with breast cancer risk (OR = 2.08 (95% CI: 1.32–3.28) *p* = 0.000779; after correction *p*_*adj*_ = 0.0350).

**Table 3:** Logistic regression and interaction analysis results for East Asian samples for SNPs with p<0.05 before FDR.

								Interaction p-value			
SNP	Gene	Alleles (Major/Minor)	Minor Allele Frequ.	OR	95% CI	*p*-value	FDR-corrected *p*-value	obesity	smoking status	exercise	ER/PR status corrected p-value
rs1800925	*IL-13*	G/A	0.167	2.08	1.32–3.28	0.000779	***0*.*0350***	0.486	0.805	0.746	0.720
rs353291	*MIR145*	A/G	0.489	0.657	0.483–0.895	0.00704	0.158	0.396	0.782	0.933	0.526
rs2032809	*BBC3/PUMA*	G/A	0.438	1.47	1.07–2.03	0.0163	0.488	0.496	0.767	0.845	0.721
rs6676805	*RFWD2*	C/G	0.310	1.48	1.06–2.08	0.0203	0.686	0.717	0.542	0.157	0.973
rs7731023	*SKP2*	A/G	0.089	0.58	0.351–0.959	0.0343	1.00	0.986	0.910	0.143	0.767
rs7236090	*BCL2*	A/G	0.457	0.713	0.515–0.986	0.0396	1.00	0.984	0.967	0.551	***0*.*0429***
rs7874234	*TSC1*	G/A	0.101	1.81	1.00–3.28	0.0418	1.00	0.982	0.930	***0*.*0281***	0.607

Among East Asian women, SNP rs7874234 (*TSC1*) (OR = 1.81 (95% CI: 1.00–3.28) *p =* 0.0418; after correction *p*_*adj*_ = 1.00) interacted with physical activity (*p* = 0.0281). When separating cases by physical activity totals, the association between rs7874234 and breast cancer risk was only seen in women with an average lifetime weekly exercise of <89.6 MET hrs (OR = 1.24 (95% CI: 1.05–1.46) *p* = 0.00909), with no association for women with >89.6 MET hrs.

Finally, rs7236090 (*BCL2*) (OR = 0.713 (95% CI:0.515–0.986) *p* = 0.0396) was differentially associated in the hormone receptor (ER/PR) classified tumour subtypes in East Asian women (*p* = 0.00612; after correction *p*_*adj*_ = 0.0429), with the strongest association seen for hormone receptor positive tumours.

We tested the *IL-13* and *TSC1* SNPs for replication of main effects by conducting lookups in genome-wide association study (GWAS) data from the Shanghai Breast Cancer Genetics Study (SBCGS) [25]. Rs1800925 in IL-13 was significantly associated with breast cancer in one of two data sets tested, as well as in an overall meta-analysis of the SBCGS data (4305 cases and 4194 controls; OR 1.12, (95% CI: 1.03–1.21) *p* = 0.011) (**Table 4**), with the same direction of association as in the Canadian data. Because exercise data was not available for the SBCGS, we were not able to test rs7874234 in *TSC1* for interaction with exercise in the replication study. No main effect for association with breast cancer risk was observed for this SNP in the SBCGS (**Table 4**).

**Table 4:** Replication results in the Shanghai breast cancer genetics study.

SNP	Gene	Alleles (Major/Minor)*	Data set	Minor Allele Frequ.	OR	95% CI	P-value
rs1800925	*IL-13*	C/T	Data1	0.162	1.09	0.97–1.24	0.164
			Data2	0.175	1.14	1.01–1.28	***0*.*03***
			Meta	0.169	1.12	1.03–1.21	***0*.*011***
rs7874234	*TSC1*	C/T	Data1	0.103	1.03	0.89–1.19	0.725
			Data2	0.097	0.91	0.79–1.05	0.181
			Meta	0.1	0.96	0.87–1.07	0.46

*Replication assays genotyped the opposite strand of those used in the discovery analysis; for both SNPs the minor allele is referred to as A in the discovery data and as T in the replication data.

## Discussion

We report an association between rs1800925 (*IL-13*) and breast cancer risk among East Asian women, which was not heterogeneous across breast cancer subtypes. *IL-13* was associated with breast cancer risk in New Mexico [26]. A previous GWAS in the SBCGS did not report this SNP as associated with breast cancer at a genome-wide significance level [25,27], nor was it associated with breast cancer survival in Chinese women [28]. In a replication by data lookup in the SBCGS GWAS data, we found that the p-value and direction of association were consistent with association of this SNP with breast cancer in East Asian women. The odds ratio for association in the larger study was smaller, 1.12 rather than 2.08, which likely means that the true effect size is quite small. Alternatively, it is possible that the effect size is larger among East Asian women in Canada, potentially because they are not identical in ethnicity to women in Shanghai, or because of different environmental contexts. Observation of this association in two studies of East Asian women may mean that the association may be real (i.e., not a false positive), although it did not reach genome-wide significance in the original GWAS in the SBCGS. This SNP has also been associated with a shorter time to recurrence among those with early stage breast cancer [29].

In the literature, the evidence for associations with the other genes we tested was inconclusive. We were unable to replicate associations with (i) *IL-6*, as previously found in 6292 cases and 8135 controls from Germany [30], 2325 cases and 2525 controls from North America [31], and 305 cases and 200 controls from Tunisia [32]; or (ii) *FAS*, as previously found in a meta-analysis of Asian populations [33] and in a study of 1053 cases and 1102 controls from New York, USA [34]. This may be due to insufficient power in our study because of smaller numbers (922 controls and 920 cases compared to other studies [30,31,34]), or because the tagSNP approach did not examine the SNP for which the association was reported [30,31]. Alternatively, for SNPs such as rs1800795 [32], which was tested with sufficient power in women of European ancestry, the difference could be attributed to population-specific genetic differences, or to a true failure to replicate a previous false positive. Consistent with our findings, other studies also found no role for the same genes we tested in studies where populations enrolled were not enriched for positive cancer family history (for example, *TNF-a* [35] or *MDM2* [36]).

The association of *CASP8* with breast cancer depended on *BRCA1/BRCA2* mutation carrier status in some studies [37], but not others [38,39]. *TP53* variants are associated with breast cancer only in *BRCA2* carriers [40], not in the general population [41]. *Pre-miR-27a* polymorphisms were associated with familial, but not sporadic, breast cancer [42]. As we do not have information for the *BRCA1* and *BRCA2* mutation status of the women in our study, and such women would likely be a minority of those in our study, we are unlikely to detect associations that only apply to mutation carriers.

We found evidence that, in East Asian women, breast cancer risk conferred by rs7874234 (*TSC1*) could be attenuated by physical activity, as women with high total physical activity scores (>89.6 MET hrs/wk) no longer had significant association for breast cancer risk. Although obesity and smoking are known to contribute to inflammation, we did not find evidence for an interaction between these factors and genetic influencers of inflammation.

Finally, we observed heterogeneity by tumour subtype for the *BCL2* SNP rs7236090, for which the minor allele was protective of breast cancer. BCL2 has been described as a favourable prognostic marker for all types of early-stage breast cancer [43]. In our dataset among East Asian women, the protective effect was strongest in women with ER/PR positive tumours, a lower risk subgroup of breast cancer.

Overall, we found evidence for associations with breast cancer risk with single SNPs in 2 genes (*RFWD2* and *TSC2)* among women of European descent, but neither remained statistically significant after correction for multiple testing. We found one SNP in *IL-13* associated with breast cancer among women of East Asian descent that remained statistically significant after multiple testing correction, and this was replicated in the SBCGS. Six other SNPs had evidence for an association in East Asian women before multiple testing correction; of these, one in *TSC1* appears to interact with physical activity.

It is important not to over-interpret the apparent negative results for many genes. We did not fully tag these genes, on average using 5 SNPs per gene and an *r*^2^ cutoff of 0.8, so there may be some unrepresented genetic variation. Even if many genes have an apparent null association with breast cancer risk, these pathways could still be important. For example, polymorphisms in microRNA binding sites, but not in the microRNAs themselves, have been associated with breast cancer [44]. Recent genome-wide association studies of breast cancer have revealed a strong enrichment for SNPs in distal transcription factor binding sites [45], which our study did not capture. Further, our relatively small sample size limited our power to detect individual SNPs with very small effects.

In conclusion, we replicated an association of SNP rs1800925 in *IL-13* with breast cancer risk among women of East Asian descent, and not among women of European descent. We also provide evidence that the *TSC1* SNP rs7874234 may interact with physical activity to influence breast cancer risk. Finally, we find that rs7236090 in *BCL2* has the strongest protective effect associated with hormone receptor positive breast cancer subtype among women of East Asian descent. These findings support the importance of considering potential environmental interactions in genetic susceptibility models, while finding an approach that balances the increased number of statistical tests conducted.

## Supporting information

S1 Table SNPs for which genotyping was attempted and whether they passed Q/C. (PDF)

S2 Table Logistic regression and interaction analysis results for European samples for SNPs with p<0.05 before FDR. (PDF)
