# Supplementary material for: Genetic variants in genes related to inflammation, apoptosis and autophagy in breast cancer risk
Source: PLoS One. 2019 Jan 2;14(1):e0209010. doi: 10.1371/journal.pone.0209010 (PMC6314637; doi:10.1371/journal.pone.0209010)
Supplement: S1 Table — (PDF) [file pone.0209010.s001.pdf]

S1 Table. SNPs for which genotyping was attempted, and whether they passed Q/C and were included in analysis.

Gene	SNP	Passed Q/C?
TP53	rs1042522	1
TP53	rs2287497	1
TP53	rs9893249	1
TP53	rs1641510	1
MDM2	rs2279744	1
MDM2	rs937282	1
pre-miRNA-27a	rs895819	0
DRAM	rs7305951	0
DRAM	rs4764678	1
DRAM	rs17032046	1
DRAM	rs7300643	1
DRAM	rs7955730	1
DRAM	rs10860821	1
DRAM	rs6539014	1
FRAP1	rs4845982	1
FRAP1	rs2295080	1
FRAP1	rs1770345	1
FKBP1A	rs1323135	1
FKBP1A	rs747611	1
FKBP1A	rs6041861	1
FKBP1A	rs9647031	1
FKBP1A	rs6041750	1
FKBP1A	rs6514364	1
FKBP1A	rs6041970	1
FKBP1A	rs1294690	1
PARK2	rs10806763	1
PARK2	rs6900416	1
PARK2	rs7764309	1
PARK2	rs2276201	1
PTEN	rs1234225	1
PTEN	rs12572106	1
PTEN	rs478839	1
PTEN	rs1234223	0
PTEN	rs1234221	1
FLJ20294	rs2902858	1
FLJ20294	rs11038910	1
ATG16L1	rs2241880	1
KIAA0831	rs11158037	1
KIAA0831	rs8013713	1
KIAA0831	rs3783653	1
KIAA0831	rs10138446	1
KIAA0226	rs7612230	1
KIAA0226	rs6583254	1
KIAA0226	rs3749249	1
KIAA0226	rs4508802	1
KIAA0226	rs6583248	1
KIAA0226	rs6787318	1
AKT1	rs2494743	1
AKT1	rs10136000	1
AKT1	rs10138227	1
AKT1	rs2498794	1
AKT1	rs2498789	1
AKT1	rs2494731	1
TSC1	rs7874234	1
TSC1	rs7858160	1
TSC1	rs1076160	1
TSC1	rs10491534	1
TSC1	rs739442	0
TSC1	rs1062218	1
TSC1	rs1050700	1
TSC1	rs11243929	1
TSC1	rs13295430	1
TSC2	rs8063461	1
TSC2	rs2516739	1
TSC2	rs8050755	1
TSC2	rs9928737	1
TSC2	rs30259	1
TSC2	rs2074969	1
TSC2	rs7187438	0
LKB1/STK11	rs35576999	1
LKB1/STK11	rs3764640	0
LKB1/STK11	rs12977299	1
LKB1/STK11	rs35369365	1

LKB1/STK11	rs12977689	1
MIR26A1	rs7372209	1
MIR30A	rs2222722	1
MIR30A	rs13202946	1
MIR125A	rs12976445	1
MIR125A	rs12975333	0
MIR125A	rs11881781	1
MIR145	rs353291	1
MIR206	rs16882131	1
MIR206	rs62408583	1
BAX	rs905238	1
BAX	rs11667229	1
BAX	rs1805419	1
BAX	rs11667351	1
BAX	rs4645878	1
BAX	rs704243	1
BBC3/PUMA	rs2032809	1
BBC3/PUMA	rs884171	1
BCL2	rs2850761	0
BCL2	rs4456611	1
BCL2	rs1026825	1
BCL2	rs7240326	1
BCL2	rs2279115	1
BCL2	rs1462129	1
BCL2	rs7236090	1
BCL2	rs7226979	1
BCL2	rs4941185	1
BCL2	rs1801018	1
BCL2	rs17759659	1
BCL2	rs7230970	1
BCL2	rs1944423	1
BCL2	rs1564483	1
BCL2	rs1982673	1
BCL2	rs10503078	1
BCL2	rs4987853	1
BECN1	rs10512488	1
BMI1	BMI1-UPSTR(	0
FAS	rs1926197	1
FAS	rs2147420	1
FAS	rs1571013	1
FAS	rs978522	1
FAS	rs982764	1
FAS	rs10509561	1
FAS	rs2234978	1
FAS	rs1159120	1
FAS	rs2234767	1
FAS	rs9658750	1
FAS	rs983751	1
FAS	rs3758483	1
MDM2	rs3730536	1
MDM2	rs1625525	1
MDM2	rs937283	1
MDM2	rs2870820	1
MDM2	rs1846402	1
MDM2	rs1695147	1
miR15a	rs9535416	1
MIRN155	rs2829803	1
MIRN155	rs928883	1
PMAIP1/NOXA	rs4286195	1
PMAIP1/NOXA	rs7240884	1
PMAIP1/NOXA	PMAIP1-x2(4[illegible]	1
PMAIP1/NOXA	rs11663656	0
PMAIP1/NOXA	rs9957673	1
PMAIP1/NOXA	rs1041978	1
RFWD2	rs617078	1
RFWD2	rs2481641	1
RFWD2	rs6676805	1
SKP2	rs7731023	1
SKP2	rs12655652	1
SKP2	rs10071838	1
SKP2	rs2362973	1
SKP2	rs17279275	1
SKP2	rs4440390	1
SKP2	rs33671	1
SKP2	rs33678	1

SKP2	rs3804439	1
TNF-alpha	rs1800629	0
TNF-alpha	rs361525	1
IL23R	rs11805303	1
IL23R	rs11209026	1
IL23R	rs7517847	1
IL23R	rs6687620	1
IL23R	rs7532161	1
IL23R	rs2201841	1
IL23R	rs1343151	1
IL23R	rs1004819	1
IL23R	rs10489629	1
IL23R	rs10789229	1
IL23R	rs7528924	1
IL23R	rs6682033	1
IL23R	rs6693831	1
IL23R	rs11209018	1
MAPK14	rs10807156	1
MAPK14	rs851027	1
MAPK14	rs3752525	1
MAPK14	rs3804454	1
MAPK14	rs10456082	1
MAPK14	rs13196204	1
MAPK14	rs851006	1
IL1B	rs16944	0
IL1B	rs1143634	1
IL1B	rs3136558	1
IL1B	rs1143633	1
CFH	rs1061170	1
IL4R	rs1801275	1
CASP8	rs1045485	1
TGFB1	rs1982073	1
PTPN22	rs2476601	1
IRF5	rs2004640	1
CTLA4	rs3087243	1
IFIH1	rs1990760	1
SEPS1	rs28665122	1
INF gamma	rs2069705	1
IL-4	rs2243248	1
IL-13	rs1800925	1
IL10	rs1800896	1
IL10	rs1800871	1
IL10	rs1800872	1
IL10	rs3024505	1
IL10	rs1554286	1
IL10	rs3024498	1
IL6	rs1800795	1
IL6	rs1800797	1
IL6	rs1800796	1
IL6	rs2069849	1
IL6	rs2069832	1
IL6	rs2069840	1
IL6	rs10242595	0
H2AFX	rs2509851	1
H2AFX	rs2509049	1
H2AFX	rs1134734	1
H2AFX	rs1064193	1
H2AFX	rs7350	1
H2AFX	rs673768	1
H2AFX	rs1804690	1
H2AFX	rs3825061	1
H2AFX	rs494048	1
H2AFX	rs1784304	0
H2AFX	rs640603	1
H2AFX	rs643788	1
H2AFX	rs571445	1
H2AFX	rs8551	1
H2AFX	rs604714	1
H2AFX	rs603826	0
H2AFX	rs649870	1
H2AFX	rs7759	1
YY1	rs4905941	1
YY1	rs1042897	1
