# Supplementary material for: Genetic variants in genes related to inflammation, apoptosis and autophagy in breast cancer risk
Source: PLoS One. 2019 Jan 2;14(1):e0209010. doi: 10.1371/journal.pone.0209010 (PMC6314637; doi:10.1371/journal.pone.0209010)
Supplement: S2 Table — (PDF) [file pone.0209010.s002.pdf]

S2 Table. Logistic Regression and Interaction Analysis Results for European Samples for SNPs with p<0.05 before FDR

SNP	Gene	OR	95% CI	*p*-value	FDR-corrected *p*-value	interaction *p*-value			ER/PR status corrected *p*-value	HER2 status corrected *p*-value
						obesity	smoking status	exercise		
rs6676805	RFWD2	0.730	0.600 - 0.889	0.00158	0.213	0.322	0.465	0.850	0.530	0.154
rs617078	RFWD2	0.799	0.681 - 0.938	0.00585	-	0.294	0.539	0.637	0.457	-
rs8063461	TSC2	0.823	0.711 - 0.963	0.0139	0.960	0.583	0.319	7.000	0.418	0.291
